# Next-Generation Metagenome Sequencing Shows Superior Diagnostic Performance in Acid-Fast Staining Sputum Smear-Negative Pulmonary Tuberculosis and Non-tuberculous Mycobacterial Pulmonary Disease

**DOI:** 10.3389/fmicb.2022.898195

**Published:** 2022-07-01

**Authors:** Peng Xu, Ke Yang, Lei Yang, Zhongli Wang, Fang Jin, Yubao Wang, Jing Feng

**Affiliations:** ^1^Department of Respiratory and Critical Care Medicine, Tianjin Medical University General Hospital, Tianjin, China; ^2^Department of Respiratory and Critical Care Medicine, The Affiliated Hospital of Inner Mongolia Medical University, Hohhot, China; ^3^Department of Respiratory and Critical Care Medicine, Shandong Second Provincial General Hospital, Jinan, China; ^4^Institute of Infectious Diseases, The Second Hospital of Tianjin Medical University, Tianjin, China

**Keywords:** bronchoalveolar lavage fluid, pulmonary tuberculosis, next-generation metagenome sequencing, diagnostic value, non-tuberculous mycobacteria

## Abstract

In this study, we explored the clinical value of next-generation metagenome sequencing (mNGS) using bronchoalveolar lavage fluid (BALF) samples from patients with acid-fast staining (AFS) sputum smear-negative pulmonary tuberculosis (PTB) and non-tuberculous mycobacterial pulmonary disease (NTM-PD). Data corresponding to hospitalized patients with pulmonary infection admitted to the hospital between July 2018 and July 2021, who were finally diagnosed with AFS sputum smear-negative PTB and NTM-PD, were retrospectively analyzed. Bronchoscopy data as well as mNGS, Xpert, AFS (BALF analysis), and T-SPOT (blood) data, were extracted from medical records. Thereafter, the diagnostic performances of these methods with respect to PTB and NTM-PD were compared. Seventy-one patients with PTB and 23 with NTM-PD were included in the study. The sensitivities of mNGS, Xpert, T-SPOT, and AFS for the diagnosis of PTB were 94.4% (67/71), 85.9% (61/71), 64.8% (46/71), and 28.2% (20/71), respectively, and the diagnostic sensitivity of mNGS combined with Xpert was the highest (97.2%, 67/71). The specificity of Xpert was 100%, while those of AFS and T-SPOT were 73.9% (17/23) and 91.3% (21/23), respectively. Further, the 23 patients with NTM-PD could be identified using mNGS, and in the population with immunosuppression, the sensitivities of mNGS, Xpert, T-SPOT, and AFS were 93.5% (29/31), 80.6% (25/31), 48.4% (15/31), and 32.3% (10/31), respectively, and the diagnostic sensitivity of mNGS combined with Xpert was the highest (100%, 31/31). The specificities of Xpert and T-SPOT in this regard were both 100%, while that of AFS was 40% (2/5). Furthermore, using mNGS, all the NTM samples could be identified. Thus, the analysis of BALF samples using mNGS has a high accuracy in the differential diagnosis of MTB and NTM. Further, mNGS combined with Xpert can improve the detection of MTB, especially in AFS sputum smear-negative samples from patients with compromised immune states or poor responses to empirical antibiotics.

## Introduction

Tuberculosis (TB), classified as pulmonary tuberculosis (PTB) or extrapulmonary tuberculosis, is an infectious disease that is caused by the *Mycobacterium tuberculosis* complex (MTBC). Specifically, PTB accounts for approximately 80% of all TB cases (WHO, 2021), and globally, the number of TB cases in 2020 was approximately 10 million, including 5.8 million new cases. Within this same year, TB also accounted for 1.5 million deaths. A greater proportion of patients with TB are adults (approximately 90%), with 8% of the total number of cases globally registered in China (WHO, 2021). Accurate and timely diagnosis of PTB is not only the basis for a successful treatment but also is key to improving the prognosis of patients and preventing the spread of the disease.

Presently, PTB diagnosis methods primarily include acid-fast staining (AFS), *Mycobacterium tuberculosis* culturing, and nucleic acid amplification (Xpert). The specificity of the culture method for the diagnosis of PTB is >99%. However, its sensitivity is approximately 80% (Lee et al., 2019). Additionally, it is time consuming (2–8 weeks), and this impedes early diagnosis (Pai et al., 2016a,b; Batyrshina and Schwartz, 2019). In contrast, AFS sputum smear microscopy is a fast and widely used TB screening method. However, its sensitivity is only approximately 50–60% (Young et al., 2008; Deshwal et al., 2019). In this regard, some authors have proposed that AFS using bronchoalveolar lavage fluid (BALF) samples can be performed in patients with negative sputum smears; however, the sensitivity of this test still needs to be improved (Shi et al., 2020; Liu et al., 2021). Further, whether using sputum or BALF smears, the major challenge associated with TB diagnosis using AFS is that a non-tuberculous mycobacteria (NTM) infection can be mistaken for an *Mycobacterium tuberculosis* (MTB) infection, resulting in patients receiving incorrect treatment. Xpert MTB/RIF assay (Cepheid, Sunnyvale, CA, United States) has been recognized by the World Health Organization (WHO) for preliminary diagnosis of suspected TB cases. However, even though its diagnostic specificity for PTB is >98%, its sensitivity for sputum smear-negative PTB is only 60%. Additionally, it does not offer the possibility of diagnosing NTM infections (Lee et al., 2019, 2020, CDC, 2019). Therefore, in clinical practice, it is challenging to distinguish MTB infections from NTM infections.

Metagenomic next-generation sequencing (mNGS) is a rapid diagnostic tool that differs from traditional microbial culture methods. mNGS identifies the possible pathogens through sequencing of nucleic acids in clinical samples, constructing biological reference databases, and rigorous sequence read mapping. This greatly improves the diagnostic ability (Doan et al., 2016; Chiu and Miller, 2019). Currently, mNGS is used for the diagnosis of various infection-related pathogens, including respiratory tract infections (Langelier et al., 2018; Xie et al., 2019), blood infections (Long et al., 2016), abscesses (Zhang et al., 2019), meningitis, and encephalitis (Guan et al., 2016; Zhao et al., 2020). mNGS can help detect intracellular pathogens and mycobacteria families (Zhang et al., 2019; Shi et al., 2020). However, only a few reports have described its use in the diagnosis of suspected PTB. The subjects included in the previous studies were AFS sputum smear-positive patients with normal immune function (Brown et al., 2015; Votintseva et al., 2017; Doyle et al., 2018; Zhou et al., 2019). To the best of our knowledge, there are only a few studies that evaluated the diagnostic value of mNGS in AFS sputum smear-negative PTB patients (Brown et al., 2015; Votintseva et al., 2017; Doyle et al., 2018; Zhou et al., 2019). We aimed to improve the diagnosis of PTB in immunocompromised patients using the mNGS approach. Conversely, whole-genome sequencing (WGS) has been used to identify *Mycobacterium abscessus* (Bryant et al., 2013; Sassi and Drancourt, 2014; Harris et al., 2015) and *Mycobacterium avium* (Operario et al., 2019), and few case reports (He et al., 2020; Taur et al., 2021) and cases (Huang et al., 2019) have described NTM determination using mNGS. More importantly, there is no previous study on the identification of MTB and NTM in patients with unexplained pulmonary infection with AFS negative sputum smear using mNGS. PTB and NTM-PD are similar in terms of clinical presentation; there have been cases of NTM-PD misdiagnosed as PTB (Maiga et al., 2012; Shahraki et al., 2015). However, there are significant differences between the two strains in treatment results and prognosis. Therefore, it is particularly important to identify NTM-PD in patients with suspected PTB during clinical diagnosis and treatment. However, in previous studies on the application value of mNGS in the diagnosis of suspected PTB, the control group was predominantly characterized by a common pathogen and was rarely identified with NTM infection. (Brown et al., 2015; Votintseva et al., 2017; Doyle et al., 2018; Zhou et al., 2019). In addition, there is no definitive conclusion on the ability of mNGS to distinguish NTM from MTB infection.

Thus, we conducted a single-center retrospective study involving hospitalized patients with suspected PTB and non-tuberculous mycobacterial pulmonary disease (NTM-PD) who were sputum smear-negative based on AFS, aiming to clarify the clinical value of mNGS in the diagnosis of AFS sputum smear-negative PTB and NTM-PD.

## Materials and Methods

### Research Design and Participants

This retrospective survey was approved by the Ethics Committee for trial registration (approval number ChiCTR1900023727) of the Tianjin Medical University General Hospital. In total, 120 patients was included in this retrospective study. The inclusion criteria were as follows: (1) patients hospitalized at the Tianjin Medical University General Hospital from July 2018 to July 2021; (2) patients with AFS-negative sputum smear and suspected PTB; (3) hospitalized patients who were eventually diagnosed with active PTB and NTM-PD; and (4) patients who underwent BALF acid-fast staining, mNGS, Xpert, and blood T-SPOT test during hospitalization.

The criteria for determining suspected PTB were as follows: (1) preliminary diagnosis based on pneumonia signs or symptoms (i.e., fever, new or increased cough, new or increased purulent sputum, chest pain, and new or increased dyspnea); (2) imaging analysis showing new infiltration and consolidation, or other lesions at the time of admission, with the new lesions not attributable to other causes (Qu and Cao, 2016); and (3) at least one antibiotic treatment administered before admission.

### Diagnostic Criteria

Mycobacterium lung diseases include PTB and NTM-PD. The diagnosis of active PTB was based on the WHO TB treatment guidelines (fourth edition) and the Chinese Tuberculosis Clinical Treatment Guidelines (2017 edition) (Committee, 2010, Electron, 2018), while the diagnosis of NTM-PD was based on the consensus of experts on the diagnosis and treatment of NTM-PD in China and the 2020 guidelines for the diagnosis and treatment of ATS/ERS/ESCMID/IDSA (Haworth et al., 2017; Association, 2020; Daley et al., 2020).

Patients were classified as immunocompromised if they met any of the following criteria (Pan et al., 2019): (1) chemotherapy or neutropenia at <1,000 cells per μL within the past 28 days; (2) corticosteroid treatment at ≥20 mg per day for 14 days; (3) immunosuppressive therapy after organ or stem cell transplantation; and (4) human immunodeficiency virus (HIV) infection.

### Specimen Collection and Processing

Experienced doctors collected BALF samples and lung biopsy specimens through bronchoscopy according to standard operating procedures (Meyer et al., 2012). During bronchoscopy, the surgeon recorded complications such as bleeding, fatal hemoptysis, arrhythmia, and death. Within 2 h after collection, lung biopsy specimens were sent to the histopathology laboratory and then processed according to standard procedures (Zhao et al., 2021). The remaining lung biopsy specimens were stored at −80°C for mNGS. Part of the BALF was used for Xpert, MTB, and microscopic smears and part was used for fungal and bacterial culture. The remaining BALF specimens were stored at −80°C for mNGS.

### Next-Generation Metagenome Sequencing

Alveolar lavage fluid samples (5 ml) and biopsy specimens (200 mg) were collected. After enrichment through centrifugation at 12,000 × *g*, at 4°C, for 10 min, 400 μl of the treated samples were mixed with glass beads and lysed using BioPrep-24 (TIANGEN BIOTECH, Beijing, China). The microbial DNA was extracted and purified using a magnetic bead method (MAGEN BIOTECH, Guangzhou, China). The concentration of the extracted nucleic acids was measured using a Qubit 4 (Thermo Fisher Scientific) fluorometer. The extracted nucleic acids were used to construct the metagenomic library using NextEra XT DNA Library Prep Kit (Illumina, San Diego, CA, United States), according to the manufacturer’s instructions. In brief, the DNA was fragmented, followed by terminal repair and primer ligation. Finally, the nucleic acids were amplified to construct a library that meets the sequencing requirements (library size: ∼300 bp). The quality of the library was evaluated using a Qubit 4 fluorometer (Thermo Fisher Scientific) and agarose gel electrophoresis. The qualified libraries with different index junction sequences were mixed at equal concentrations. High-throughput sequencing was performed using the Illumina NextSeq 550 DX sequencing platform (sequencing strategy: SE75). During trimming, the low-quality bases, and short sequences (<35 bp) were removed, the remaining clean reads were compared with the human genome reference sequence (version: GRCh38), and Bowtie2 (BWA) was used to remove human-related reads. The remaining reads were further compared with the pathogenic microorganism database^1^, and the detected sequence number (reads) for pathogenic microorganisms was obtained. According to the interpretation standard of pathogen pathogenicity: (1) *Mycobacterium tuberculosis* was considered positive when at least one read was aligned to the reference genome at the species or genus level and (2) NTM was defined as positive when the mapping reading (genus or species level) was at the top 10 of the bacterial list; (3) for bacteria other than mycobacteria, viruses, and parasites, the top 10 bacteria in each category were selected, and suspicious pathogens were reported in combination with clinical characteristics (Miao et al., 2018).

### Statistical Analysis

Continuous variables corresponding to the two groups were compared using an independent sample *t*-test or Mann–Whitney *U* test, and the classified treatments were compared using the chi-square test or the Fisher exact probability method. All statistical analyses were performed using SPSS software v25.0 (IBM, Armonk, NY, United States) and GraphPad Prism 8.0, and statistical significance was defined as *p* < 0.05.

## Results

### Baseline Information of Subjects

**FIGURE 1:**
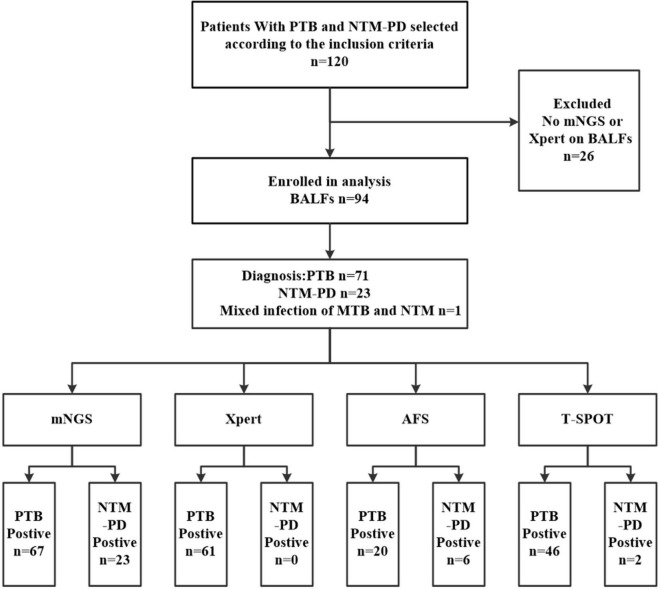
Technology roadmap.

As shown in Figure 1, between July 2018 and July 2021, 120 hospitalized patients with undetermined causes of pulmonary infection were selected and finally diagnosed with PTB or NTM-PD. Notably, all the patients were AFS sputum smear-negative. However, 26 patients without BALF sample analysis data based on mNGS or Xpert were excluded. Thus, 94 cases (71 PTB cases and 23 NTM-PD cases) were ultimately selected.

As shown in Table 1, the NTM-PD group, which predominantly consisted of female patients (17/23, 73.9%), showed a higher incidence of structural lung disease (14/23, 60.9%), especially bronchiectasis with non-cystic fibrosis than the PTB group (9/71, 39.1%). Further, the imaging results showed a higher incidence of bronchiectasis with non-cystic fibrosis (9/23, 39.1%) and emphysema (4/23, 17.4%) in the NTM-PD group than in the PTB group (*p* > 0.05). However, there were no significant differences between them with respect to age, symptoms, immune dysfunction, imaging features, histopathology, CRP, PCT, ferritin, white blood cell count, and albumin level. Additionally, 36 cases (38.3%), including 31 in the PTB group and 5 in the NTM-PD group, were immunocompromised to some extent.

**TABLE 1:** Baseline characteristics and clinical indices of PTB and NTM-PD subjects.

Characteristics	Total (*n* = 94)	PTB (*n* = 71)	NTM-PD (*n* = 23)	*p*
**Age, years**	52.02 ± 17.81	50.55 ± 18.82	58.13 ± 12.96	0.076
**Gender, *n* (%)**				
Male	44 (46.8)	38 (53.0)	6 (26.1)	0.026
Female	50 (53.2)	33 (47.0)	17 (73.9)	
**Symptoms, *n* (%)**				
Fever	47/94 (50.0)	37/71 (52.1)	10/23 (43.5)	0.472
Cough	64/94 (68.1)	48/71 (67.6)	16/23 (69.6)	0.861
Expectoration of phlegm	43/94 (45.7)	33/71 (46.5)	10/23 (43.5)	0.802
Hemoptysis	15/94 (16.0)	11/71 (15.5)	4/23 (17.4)	1.000
Chest pain	10/94 (10.6)	8/71 (11.3)	2/23 (8.7)	1.000
Dyspnea	7/94 (7.4)	5/71 (7.0)	2/23 (8.7)	1.000
**Symptom duration, days**	175.54 ± 199.09	170.77 ± 179.96	190.262 ± 53.39	0.245
**Complications, n (%)**				
Interstitial lung disease	2/94 (2.1)	0/71 (0.0)	2/23 (8.7)	0.058
Bronchiectasis with non-cystic fibrosis	16/94 (17.0)	7/71 (9.9)	9/23 (39.1)	0.003
Chronic obstructive pulmonary disease	7/94 (7.4)	4/71 (5.6)	3/23 (13.0)	0.185
Diabetes	16/94 (17.0)	13/71 (18.3)	3/23 (13.0)	0.791
Connective tissue disease	8/94 (8.5)	7/71 (9.9)	1/23 (4.3)	0.694
Kidney disease	3/94 (3.2)	3/71 (4.2)	0/23 (0.0)	1.000
Hematologic diseases	4/94 (4.3)	2/71 (2.8)	2/23 (8.7)	0.250
Hematological malignant tumor	19/94 (20.2)	18/71 (25.4)	1/23 (4.3)	0.060
Hematopoietic stem cell transplantation	10/94 (10.6)	9/71 (12.7)	1/23 (4.3)	0.461
Malignant tumor of the lung	2/94 (2.1)	2/71 (2.8)	0/23 (0.0)	1.000
**Combined with immunosuppression, *n* (%)**	36/94 (38.3)	31/71 (43.7)	5/23 (21.7)	0.060
**Combined with structural lung disease, *n* (%)**	29/94 (30.9)	15/71 (21.1)	14/23 (60.9)	<0.001
**Imaging features, *n* (%)**				
Tree-in-bud	42/94 (44.7)	28/71 (39.4)	14/23 (60.9)	0.072
Consolidation	47/94 (50.0)	36/71 (50.7)	11/23 (47.8)	0.810
Ground glass opacity	28/94 (29.8)	20/71 (28.2)	8/23 (27.3)	0.547
Nodule shadows	45/94 (47.9)	31/71 (43.7)	14/23 (60.9)	0.151
Cavitary lesions	25/94 (26.6)	19/71 (26.8)	6/23 (26.1)	0.949
Emphysema	6/94 (6.4)	2/71 (2.8)	4/23 (17.4)	0.046
Bronchiectasis with non-cystic fibrosis	20/94 (21.3)	11/71 (15.5)	9/23 (39.1)	0.030
**Pathological features of biopsy tissue, *n* (%)**				
Granulomatous lesion	34/94 (36.2)	26/71 (36.7)	8/23 (34.8)	0.873
Necrotizing granuloma	18/94 (19.4)	17/71 (23.9)	1/23 (4.3)	0.077
Necrosis	33/94 (35.1)	27/71 (38.0)	6/23 (26.1)	0.297
Acute and chronic non-specific inflammation	22/94 (24.0)	16/71 (20.3)	6/23 (26.1)	0.727
**Laboratory index**				
CRP, ng/dL	1.07 (0.29–3.57)	1.09 (0.30–3.68)	1.02 (0.24–2.60)	0.704
Ferritin, ng/mL	156.80 (87.56–457.04)	198.16 (92.32–567.81)	102.35 (85.49–189.90)	0.081
PCT-positivity, *n* (%)	3/94 (3.2)	2/71 (2.8)	1/23 (4.3)	1.000
Albumin, g/L	35.91 ± 5.29	36.35 ± 5.42	34.65 ± 4.80	0.187
Leukocyte, 10^9^/L	6.40 ± 3.03	6.10 ± 3.15	7.24 ± 2.55	0.122
Types of antibiotics used, *n* (%)				
Antifungal agents	22/94	21/71	1/23	NA
Fluoroquinolones	56/94	44/71	12/23	NA
β-Lactams and enzyme containing inhibitors	56/94	43/71	13/23	NA
Carbapenems	14/94	13/71	1/23	NA
Linezolid	12/94	11/71	1/23	NA
Sulfonamides	5/94	3/71	2/23	NA
Macrolides	7/94	3/71	4/71	NA
Tigecycline	2/94	2/71	0/23	NA

*Data are expressed as the mean ± standard deviation, median (P25–P75), and number of cases (%). Statistical methods, t-test, corrected t-test, and Mann–Whitney test were used for the comparison of the measurement data corresponding to the two groups, and the Chi^2^ test and Fisher probability method were used for the comparison of the counting data corresponding to the two groups. NA, not applicable.*

### Comparison of the Detection Performances of Next-Generation Metagenome Sequencing, Xpert, Acid-Fast Staining (Bronchoalveolar Lavage Fluid Samples), and T-SPOT (Sera Samples) to Distinguish *Mycobacterium tuberculosis* From Non-tuberculous Mycobacteria

**FIGURE 2:**
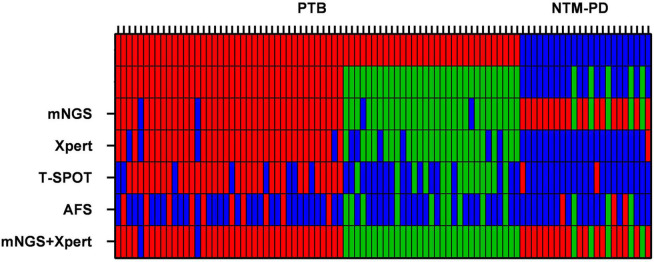
Heat maps indicating the performances of mNGS, Xpert, AFS (BALF samples), and T-SPOT (sera samples) in the diagnosis of MTB and NTM. The red bars indicate that MTB and NTM were detected correctly. The green bars indicate that MTB and NTM were detected correctly in immunocompromised patients.

**FIGURE 3:**
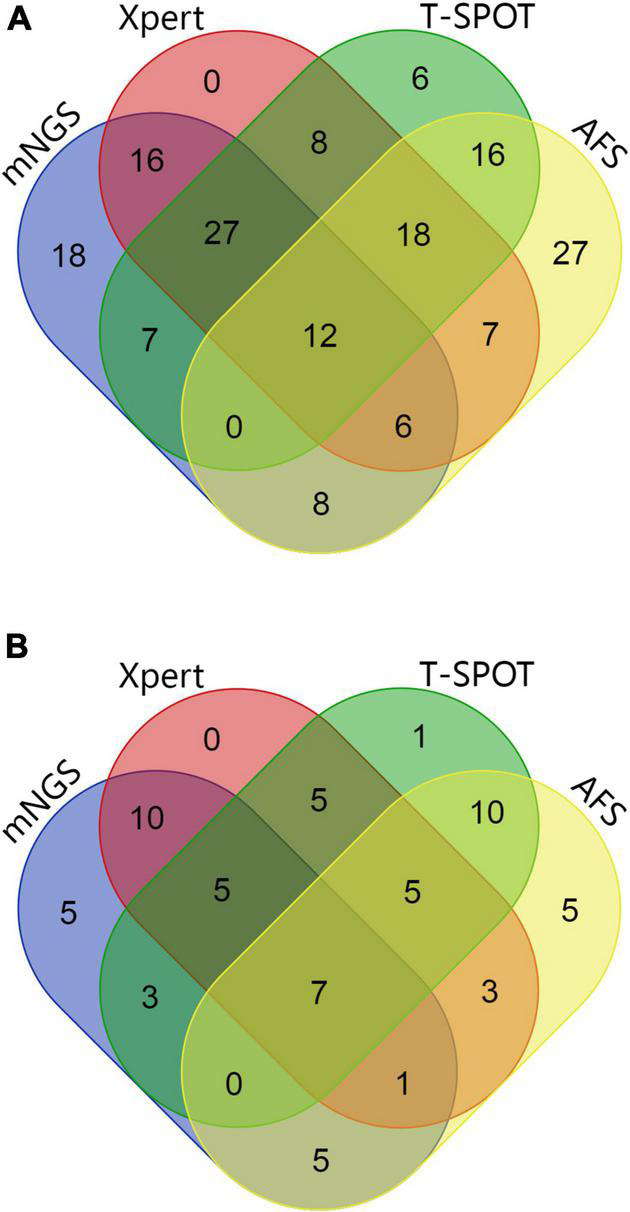
**(A)** Wayne diagram showing the evaluation of the performances of mNGS, Xpert, AFS (BALF samples), and T-SPOT (sera samples) with respect to the detection of MTB and NTM. **(B)** Wayne diagram showing the evaluation of the performances of mNGS, Xpert, AFS (BALF samples), and T-SPOT (sera samples) in immunocompromised patients for the detection of MTB and NTM.

As shown in Table 2 and Figures 2, 3A, out of the 71 patients with PTB, based on the analysis of BALF samples, 67 cases were detected using mNGS, resulting in a sensitivity of 94.4%, followed by Xpert, with a sensitivity of 85.9% (61/71), which was significantly higher than that of the conventional methods T-SPOT 64.8% (46/71) and AFS 28.2% (20/71), and the difference was statistically significant (*p* < 0.001). However, there was no significant difference in sensitivity between mNGS and Xpert (*p* > 0.05). When the two rapid detection methods mNGS and Xpert were combined, 69 cases of MTB were detected with the highest sensitivity (97.2%), which was statistically significant compared with that of the conventional method (*p* < 0.001). Notably, for the NTM-PD group, the specificity of Xpert was 100%, implying that all the patients with NTM-PD were Xpert-negative. However, the specificities of AFS and T-SPOT were 73.9% (17/23) and 91.3% (21/23), respectively. These results indicated that neither AFS only nor T-SPOT only can be used to diagnose PTB. Further, mNGS showed the ability to recognize both MTB and NTM, indicating its superior diagnostic performance relative to Xpert, AFS, and T-SPOT. Notably, the 23 patients with NTM-PD in this study could all be identified using mNGS, indicating that the specificity of mNGS was the same as that of Xpert. The specificity was statistically significant compared with that of conventional methods (*p* < 0.001).

**TABLE 2:** Comparison of the detection performances of mNGS, Xpert, AFS (BALF samples), and T-SPOT (sera samples) in all patients with *Mycobacterium tuberculosis* infection and immunocompromised patients.

Method	MTB (In all patients)		MTB (In immunocompromised patients)	
		
	Sensitivity (95% CI, n/N)	Specificity (95% CI, n/N)	Sensitivity (95% CI, n/N)	Specificity (95% CI, n/N)
mNGS	94.4 (0.889–0.999, 67/71)	100 (0.999–1, 23/23)	93.5 (0.844–1, 29/31)	100 (0.999–1, 5/5)
Xpert	85.9 (0.776–0.942, 61/71)	100 (0.999–1, 23/23)	80.6 (0.660–0.954, 25/31)	100 (0.999–1, 5/5)
AFS	28.2*^#&^ (0.175–0.389, 20/71)	73.9*^#&^ (0.545–0.933, 17/23)	32.3*^#&^ (0.148–0.497, 10/31)	40 (0.28–1, 2/5)
T-SPOT	64.8*^#[*$*]^ (0.534–0.762, 46/71)	91.3 (0.789–1, 21/23)	48.4*^#[*$*]^ (0.298–0.670, 15/31)	100 (0.999–1, 5/5)
mNGS + Xpert	97.2^&[*$*]^ (0.932–1, 69/71)	100^&^ (0.999–1, 23/23)	100^&[*$*]^ (0.999–1, 31/31)	100 (0.999–1, 5/5)
*p*(χ^2^)	<0.001	<0.001	<0.001	0.22

*Compared with mNGS p < 0.05, # compared with Xpert p < 0.05, & compared with AFS p < 0.05, and $ compared with T-SPOT p < 0.05.

Immunocompromised hosts are prone to disseminated infection, which increases mortality rate, after PTB or NTM-PD (Ratnatunga et al., 2020). Given that the non-specific symptoms, signs, and imaging findings corresponding to TB often overlap with those of other pulmonary bacterial infections, the use of conventional antibiotics leads to a low diagnosis performance for the traditional diagnostic methods, leading to prolonged disease and poor therapeutic effects (Mukhopadhyay and Gal, 2010; Nachiappan et al., 2017). mNGS is a molecular method that can be used to detect pathogen DNA. Therefore, we performed subgroup analysis and selected individuals with low immunity for this study. As shown in Table 1, 36 immunocompromised patients were included in this study. Among them, 16 were treated with corticosteroids at doses ≥20 mg/day for connective tissue disease and other diseases for more than 14 days, 9 had neutropenia (<1,000 cells per μL) due to hematological malignancies, 10 received immunosuppressive therapy after hematopoietic stem cell transplantation, and 2 with lung malignant tumors received maintenance chemotherapy within 28 days.

Additionally, as shown in Table 2 and Figures 2, 3B, based on BALF analysis, 29 cases of MTB were detected using mNGS, which showed a diagnostic sensitivity of 93.5%, followed by Xpert, with a sensitivity of 80.6% (25/31), while the sensitivities of T-SPOT and AFS were 48.4% (15/31) and 32.3% (10/31), respectively. Notably, the diagnostic sensitivities of Xpert and T-SPOT were reduced in this subgroup. However, the sensitivity of mNGS was not significantly different from that of Xpert (*p* > 0.05). After mNGS and Xpert were combined, all MTB cases were detected, and the diagnostic sensitivity (100%) was the highest, which was statistically significant compared with that of the conventional method (*p* < 0.001). This indicates that the combination of mNGS and Xpert can significantly improve the diagnostic sensitivity for PTB in immunocompromised populations. For the NTM-PD groups, the specificities of Xpert and T-SPOT were both 100%, and the specificity of AFS was 40% (2/5). These findings indicated that AFS only and T-SPOT only cannot be used for the diagnosis of PTB in immunocompromised patients. It is worth noting that there were five patients with NTM-PD in this subgroup, and they all could be identified using mNGS.

Our findings also indicated that using BALF samples, MTB was not detected in 4 out of the 71 patients with PTB using mNGS; however, when mNGS was performed using bronchoscopic lung biopsy samples, MTB could be diagnosed in these 4 patients.

### Identification of Non-tuberculous Mycobacteria Strains and Mixed Infection in Bronchoalveolar Lavage Fluid Samples Using Next-Generation Metagenome Sequencing

The strains from the 23 patients in the NTM-PD group in this study were identified using mNGS. Among the patients, there were 12 cases of *Mycobacterium intracellulare*, five cases of *Mycobacterium avium*, two cases of *Mycobacterium kansasii*, two cases of *Mycobacterium abscessus*, one case of *Mycobacterium xenopi*, and 1 case of *Mycobacterium scrofulaceum.*

**FIGURE 4:**
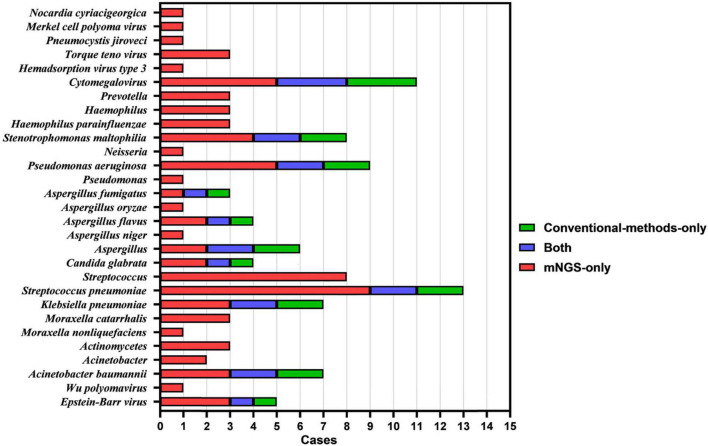
Histogram showing the results of mNGS and conventional methods in identifying mixed infection in BALF specimens of patients with PTB and NTM-PD.

**FIGURE 5:**
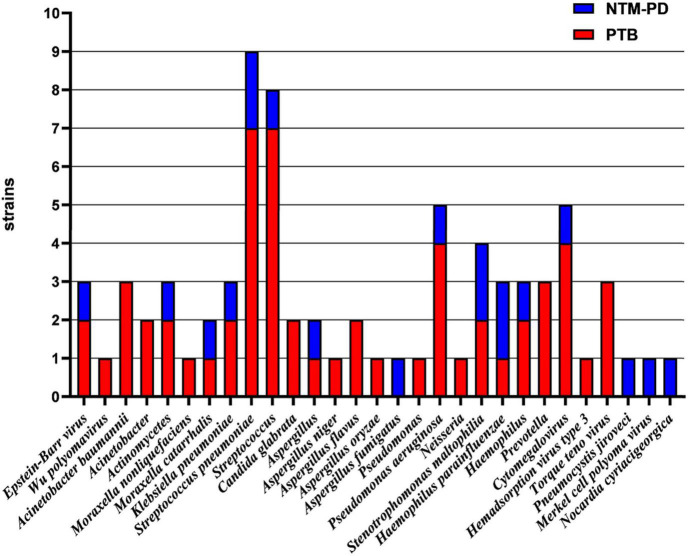
Identification of strains in BALF samples from patients with PTB and NTM-PD complicated with co-infection using mNGS.

As shown in Figures 4, 5, mNGS could also identify other mixed-pathogen infections (including bacteria, fungi, and viruses). Among the 94 patients included in our study, 50 cases were complicated by other pathogenic bacterial infections, and most of these cases were complicated with immunosuppression and structural lung disease. Further, we compared mNGS with routine clinical detection methods (BALF bacterial and fungal culture, bacterial and fungal microscopic smears, serum *Aspergillus* IgG antibody, GM test, *Cryptococcus* antigen detection, respiratory syncytial virus antibody, *Cytomegalovirus* antibody, adenovirus antibody, EB virus antibody, and human herpesvirus type I and type II antibodies). All common bacteria, including *Haemophilus parainfluenzae*, *Klebsiella pneumoniae*, *Streptococcus pneumoniae*, *Pseudomonas aeruginosa*, *Acinetobacter baumannii*, and *Stenotrophomonas maltophilia*, were detected by mNGS, with the sensitivity being significantly higher than that of BALF routine bacterial culture and microscopic smear examinations (34.5%, 10/29). Further, six cases, all of which were detected using mNGS, were complicated by fungal infection (*Aspergillus spp.*). One case of *Aspergillus fumigatus* infection was detected using the BALF fungal culture method, and three cases were positive based on fungal serum immunoassay. Furthermore, mNGS could also detect one case of *Pneumocystis carinii*, one case of *Legionella pneumophila*, one case of St. George *Nocardia*, and three cases of the genus *Actinomyces spp.*, which could not be detected using the conventional pathogen detection methods. Ten patients with viral infections, including *Circovirus*, *Cytomegalovirus*, parainfluenza virus, and polyomavirus, were also detected using mNGS. Three cases of *Cytomegalovirus* antibody (IgM and IgG) and one case of EB virus antibody (IgM) were detected using the traditional etiological methods, while others were not detected.

We also found that 44% (*n* = 22) of PTB patients with co-infection were complicated with one microbial infection, 22% (*n* = 11) with two microbial infections, 10% (*n* = 5) with three microbial infections, and 2% (*n* = 1) with five microbial infections. Among NTM-PD patients with co-infection, the proportions of patients co-infected with one, two, three, and four microorganisms were 10% (*n* = 5), 6% (*n* = 3), 4% (*n* = 2), and 2% (*n* = 1), respectively.

## Discussion

In this study, we comprehensively evaluated the performance of mNGS for the etiological diagnosis of suspected PTB patients with negative sputum smear. In the diagnosis of PTB, the diagnostic sensitivity of mNGS (94.4%) was higher than those of Xpert (85.9%) and the conventional diagnostic methods T-SPOT (64.8%) and AFS (28.2%). The diagnostic sensitivity of mNGS combined with Xpert (97.2%) was the highest, suggesting that the combination of mNGS and Xpert can improve the diagnosis of PTB, which is consistent with other reports (Zhou et al., 2019; Shi et al., 2020; Liu et al., 2021). However, the sensitivity of mNGS in this study was not significantly different from that of Xpert. Thus, mNGS is not superior to Xpert in the detection of MTB, consistent with the conclusion of other studies (Zhou et al., 2019; Shi et al., 2020). However, compared with these three detection methods, mNGS has a prominent advantage in that it can also identify NTM, which is difficult for culture-based methods to detect, because some NTM species require a time-consuming procedure to detect and some are difficult to culture (Kim et al., 2018). Since our patients used antibiotics before they were enrolled, we believe that mNGS has a higher accuracy than conventional diagnostic methods in detecting MTB and NTM in BALF specimens. Therefore, its use in the diagnosis of TB using BALF samples is a reliable method to distinguish MTB from NTM.

The importance of our study lies in the fact that this patient population lacks specific clinical symptoms, and their imaging features can be easily mistaken for pulmonary infection. Thus, patients with compromised immune function are confused with the original diseases or are considered to be infected with multiple pathogens if the clinical features of TB change. This can lead to missed diagnoses, misdiagnoses, and increased TB-related deaths (Miller et al., 2000; Liam et al., 2006). However, we found that the diagnostic sensitivity of Xpert and T-SPOT was also decreased in the immunocompromised subgroups, and Xpert was prone to false negative results, consistent with other studies (Lee et al., 2019, 2020). The combination of mNGS and Xpert can significantly improve the diagnostic sensitivity for PTB in immunocompromised populations, which has not been mentioned in other studies on the detection of *Mycobacterium tuberculosis* by mNGS and Xpert.

Studies have shown that the clinical manifestations of PTB and NTM-PD are similar and that they can have common CT manifestations in imaging, including tree bud-like signs, bronchiectasis, and cavities; moreover, necrotizing granulomas can represent histopathological features of both diseases (Mukhopadhyay, 2011; Nachiappan et al., 2017), which are consistent with the results of our study. However, in the present study, NTM was detected using mNGS, revealing the feasibility and unique advantages of mNGS in distinguishing MTB from NTM. The risk of NTM-PD was significantly higher (*P* < 0.05) in patients with structural pulmonary disease (14/23, 60.9%), especially bronchiectasis without cystic fibrosis (9/23, 39.1%), compared to that for PTB; this was consistent with the results of previous study (Cowman et al., 2019). Our study included 14 structural lung diseases in 23 patients with NTM-PD. For patients with structural or inflammatory lung diseases, such as non-cystic fibrobronchiectasis, chronic obstructive pulmonary disease, or interstitial lung disease, the accurate diagnosis of NTM infection is critical because these emerging pathogens can lead to an accelerated decline in lung function and fatal complications (Floto et al., 2016).

Additionally, it has been reported that patients with impaired immune function and structural lung disease are more susceptible to fungal and other microbial infections after PTB and NTM-PD (Wu and Holland, 2015; Zeng et al., 2020). Specifically, when an immune-impaired host has a mixed infection of pulmonary infiltration, the successful diagnosis of co-existing opportunistic infections using conventional diagnostic methods is challenging (Pan et al., 2019). Further, the use of antibiotics decreases the diagnostic rate of conventional diagnostic methods to a low level. In our study, 46% of the patients with co-infection were infected with more than two types of microorganisms. Our findings also indicated that the analysis of BALF samples using mNGS can lead to the simultaneous detection of mixed infections by bacteria, fungi, viruses, and mycobacteria, which is consistent with other reports (Pan et al., 2019; Xie et al., 2019; Zhao et al., 2021). The results of our study suggested that mNGS is a good diagnostic method for patients with pulmonary infections who have poor responses to empirical antibiotics and are AFS sputum smear-negative. mNGS also offers the possibility to identify not only MTB and NTM but also other mixed infections. Further, it is also suitable for application in immunocompromised patients with complex infections. This not only indicates an improvement in diagnostic ability in immunocompromised people with acute lower respiratory tract infections (LRTI) but also can help clinicians evaluate patients more comprehensively and prescribe a more effective treatment in time.

In our study, MTB was not detected by mNGS in four BALF specimens, although it was discovered in the four bronchoscopic lung biopsies from these patients. It has been reported that the diagnostic rate of mNGS in lung biopsy is higher than that in BALF (Mondoni et al., 2017), probably because the bacterial load in diseased tissue is higher than that in the sputum and BALF. These results suggest that we can use mNGS to analyze lung tissues from patients who are highly suspected of PTB, but whose mNGS results are negative based on the analysis of BALF.

Although some encouraging results were obtained in this study, some limitations were also noted. First, due to the relatively high cost, the widespread clinical application of mNGS may be under restrictions (Lee and Pai, 2017; Lee et al., 2019). Second, diagnosis of infections using invasive techniques, such as bronchoalveolar lavage, may lead to further respiratory deterioration in patients with hypoxemia (Bauer et al., 2019; Azoulay et al., 2020), thus the collection of BALF samples is limited. Third, the sample size was relatively small, as this was a single-center retrospective study. Therefore, a multicenter prospective study with a larger sample size is necessary to further confirm these findings.

In summary, the findings of this study indicate that the analysis of BALF samples using mNGS has a high accuracy in the differential diagnosis of MTB and NTM. Further, mNGS combined with Xpert can improve the detection of MTB, especially in AFS sputum smear-negative samples from patients with compromised immune states or poor responses to empirical antibiotics. Therefore, we believe that mNGS is a powerful tool for identifying pathogenic microorganisms, and further investigations are required on this aspect.

## Data Availability Statement

The data presented in the study are deposited in the NCBI and National Genomics Data Center repository, accession numbers: PRJNA847681, PRJNA848420, and PRJCA010004, respectively.

## Ethics Statement

The studies involving human participants were reviewed and approved by the Ethics Review Committee of Tianjin Medical University General Hospital. Written informed consent to participate in this study was provided by the participants’ legal guardian/next of kin.

## Author Contributions

YW and JF contributed to the research design and revision of the manuscript. PX and KY drafted the research protocol, analyzed the results, and drafted the manuscript. LY, ZW, and FJ contributed to the data analysis and data deposition in an acceptable repository. All authors approved the submitted version and agreed to be responsible for all aspects.

## Conflict of Interest

The authors declare that the research was conducted in the absence of any commercial or financial relationships that could be construed as a potential conflict of interest.

## Publisher’s Note

All claims expressed in this article are solely those of the authors and do not necessarily represent those of their affiliated organizations, or those of the publisher, the editors and the reviewers. Any product that may be evaluated in this article, or claim that may be made by its manufacturer, is not guaranteed or endorsed by the publisher.

## Footnotes

1
ftp://ftp.ncbi.nlm.nih.gov/genomes/
